# Postoperative Atypical Hemolytic Uremic Syndrome Associated with Complement C3 Mutation

**DOI:** 10.1155/2014/784943

**Published:** 2014-11-09

**Authors:** Eiji Matsukuma, Atsushi Imamura, Yusuke Iwata, Takamasa Takeuchi, Yoko Yoshida, Yoshihiro Fujimura, Xinping Fan, Toshiyuki Miyata, Takashi Kuwahara

**Affiliations:** ^1^Department of Pediatrics, Gifu Prefectural General Medical Center, 4-6-1 Noishiki, Gifu 500-8717, Japan; ^2^Department of Pediatric Cardiovascular Surgery, Gifu Prefectural General Medical Center, 4-6-1 Noishiki, Gifu 500-8717, Japan; ^3^Department of Blood Transfusion Medicine, Nara Medical University, 840 Shijyou-cho, Kashihara, Nara 634-8521, Japan; ^4^Department of Molecular Pathogenesis, National Cerebral and Cardiovascular Center, 5-7-1 Fujishirodai, Suita, Osaka 565-8565, Japan; ^5^Department of Pediatric Cardiology, Gifu Prefectural General Medical Center, 4-6-1 Noishiki, Gifu 500-8717, Japan

## Abstract

Atypical hemolytic uremic syndrome (aHUS) can be distinguished from typical or Shiga-like toxin-induced HUS. The clinical outcome is unfavorable; up to 50% of affected patients progress to end-stage renal failure and 25% die during the acute phase. Multiple conditions have been associated with aHUS, including infections, drugs, autoimmune conditions, transplantation, pregnancy, and metabolic conditions. aHUS in the nontransplant postsurgical period, however, is rare. An 8-month-old boy underwent surgical repair of tetralogy of Fallot. Neurological disturbances, acute renal failure, thrombocytopenia, and microangiopathic hemolytic anemia developed 25 days later, and aHUS was diagnosed. Further evaluation revealed that his complement factor H (CFH) level was normal and that anti-FH antibodies were not detected in his plasma. Sequencing of his CFH, complement factor I, membrane cofactor protein, complement factor B, and thrombomodulin genes was normal. His ADAMTS-13 (a disintegrin-like and metalloprotease with thrombospondin-1 repeats 13) activity was also normal. However, he had a potentially causative mutation (R425C) in complement component C3. Restriction fragment length polymorphism analysis revealed that his father and aunt also had this mutation; however, they had no symptoms of aHUS. We herein report a case of aHUS that developed after cardiovascular surgery and was caused by a complement C3 mutation.

## 1. Introduction

Thrombotic microangiopathy (TMA) is a clinical pathologic disorder characterized by the presence of microthrombi in multiple organ systems, including the kidneys and brain. Peripheral blood smears show fragmented red blood cells and thrombocytopenia [1]. TMA forms the pathophysiologic basis of several clinical syndromes, including hemolytic uremic syndrome (HUS); thrombotic thrombocytopenic purpura (TTP); and hemolysis, elevated liver enzymes, and low platelets (HELLP) syndrome [2]. HUS and TTP were previously considered to be part of a single spectrum of TMA. However, recent research and chemical analysis of patients' serum have indicated that HUS and TTP are separate entities with distinct pathogenetic processes [1]. Although HUS occurs infrequently, it is the most common TMA in the pediatric population. About 90% of cases in children are associated with Shiga-like toxin produced mainly by* Escherichia coli *0157:H7,* Shigella dysenteriae* type 1, and other pathogens [3]. Atypical HUS (aHUS), which can be distinguished from typical or Shiga-like toxin-induced HUS, may occur secondary to infections, malignancies, drugs, pregnancy, and autoimmune disease [3].

aHUS can be sporadic or familial. More than half of patients with aHUS exhibit genetic loss-of-function mutations of regulators (complement factor H (CFH), complement factor I (CFI), membrane cofactor protein (MCP), and thrombomodulin (THBD)) [4–7]. Additionally, gain-of-function mutations of key complement component C3 and complement factor B (CFB) [8, 9] have been found to predispose to aHUS.

However, the pathophysiology of TTP, which has been largely elucidated in recent years, involves an imbalance between the levels of von Willebrand factor and its cleaving protease, a disintegrin-like and metalloprotease with thrombospondin-1 repeats 13 (ADAMTS-13). This imbalance leads to the presence of large multimers of von Willebrand factor, which then bind platelets and form thrombi in various organs. Low activity of the cleaving protease has been noted in adults with antibodies to ADAMTS-13 [10]. Congenital defects in the ADAMTS-13 gene lead to low levels of the protease; this is the most common cause of TTP in children.

Multiple conditions have been associated with aHUS, including infections, certain drugs, autoimmune conditions, transplantation, pregnancy, and metabolic conditions [11, 12]. However, aHUS occurring in the nontransplant postsurgical period has rarely been reported.

## 2. Case Presentation

**Figure 1:**
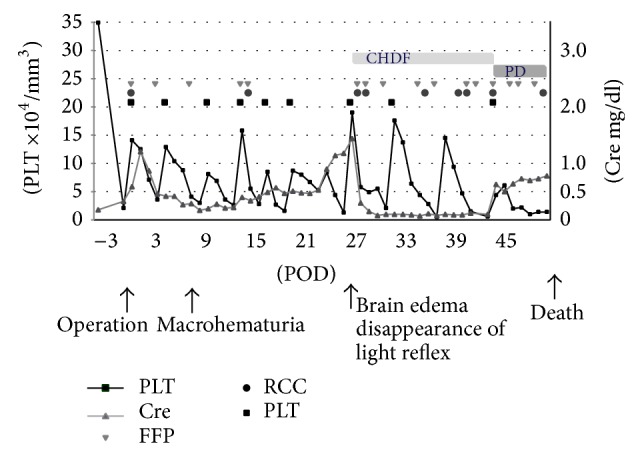
Laboratory findings during the clinical course.

An 8-month-old boy was referred to the Department of Pediatric Cardiovascular Surgery in our center for surgical repair of tetralogy of Fallot. His initial preoperative blood count and complete chemistry results were normal. His complement levels were not examined. The patient was transferred to the pediatric intensive care unit for observation and further management. On postoperative day 1, he was found to have anemia with a hemoglobin level of 10.6 g/dL and thrombocytopenia with a platelet count of 21,000/mm^3^ (Table 1(a)). The patient's prothrombin time-international normalized ratio (PT-INR) and activated partial thromboplastin time (APTT) were prolonged at 1.47 and 30.10 seconds, respectively. The patient was diagnosed with disseminated intravascular coagulation (DIC) and treated with fresh frozen plasma (FFP) and a platelet transfusion. Macrohematuria developed on postoperative day 8, and FFP and platelet transfusions were performed repeatedly to control his bleeding. However, his renal function and consciousness level only temporarily improved; disturbance of consciousness and renal dysfunction redeveloped on postoperative days 16 to 24 (Figure 1).

On postoperative day 25, physical examination demonstrated a blood pressure of 90/57 mmHg and a pulse of 105/min. Laboratory examination showed only mild anemia with a hemoglobin level of 11.5 g/dL as well as thrombocytopenia with a platelet count of 13,000/mm^3^. Schizocytes were observed in the patient's blood smear, and a low haptoglobin level (<10 mg/dL) was noted. The patient's PT-INR and APTT were prolonged at 2.33 and 45.0 seconds, respectively. Blood chemistry results disclosed renal failure, with a creatinine and blood urea nitrogen level of 1.18 and 108.00 mg/dL, respectively. His complement levels (reference ranges) were as follows: C3, 40 mg/dL (69–128 mg/dL); C4, 12.7 mg/dL (14–36 mg/dL); and CH50, 19.5 U/mL (25–50 U/mL). His other blood chemistry data were as follows: total protein, 4.9 g/dL; albumin, 2.8 g/dL; total bilirubin, 2.07 mg/dL; serum aspartate aminotransferase, 126 IU/L; and serum alanine aminotransferase, 8 IU/L (Tables 1(a) and 1(b)). Urinalysis showed proteinuria and hematuria. The patient had neither diarrhea nor bloody stool. Stool culture results were negative for* E. coli* O157. According to the above data and the patient's neurological disturbances, acute renal failure, thrombocytopenia, and microangiopathic hemolytic anemia, TMA (most likely aHUS) was considered as the working diagnosis. On postoperative day 26, brain CT was performed to identify the cause of the persistent disturbance of consciousness and showed severe, extensive brain edema. Furthermore, pupillary light reflex deficits were observed. The patient was not expected to recover without neurological sequelae. Therefore, continuous hemodiafiltration, peritoneal dialysis, and plasma transfusion were performed as conservative therapy. The patient died on postoperative day 50.

Further evaluation revealed that his CFH level was normal, and a hemolytic assay using the patient's serum and that of normal controls showed no significant difference. Anti-FH antibodies were not detected in the patient's plasma. Sequencing of the CFH, CFI, MCP, CFB, and THBD genes was normal. His ADAMTS-13 activity was also normal (Table 1(b)). However, he had a potentially causative mutation (R425C) in the b-chain of C3 in exon 12. This finding confirmed that the patient had aHUS caused by a C3 gene mutation.

**Figure 2:**
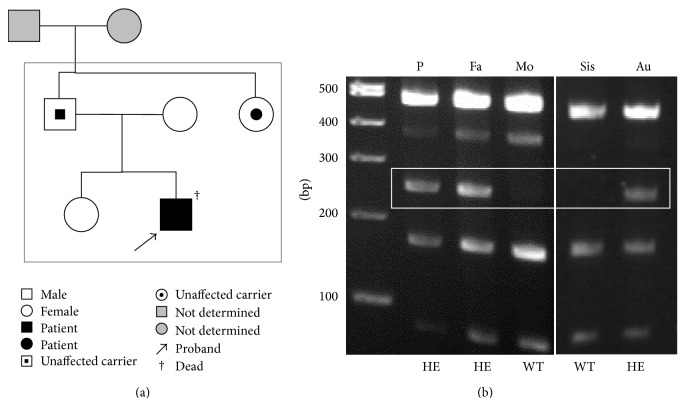
(a) The pedigree of the patient. (b) RFLP analysis. P: proband, Fa: father, Mo: mother, Sis: sister, Au: aunt, WT: wild type, and HE: heterozygote.

The patient's parents gave consent for C3 gene analysis in the patient, his elder sister, and his aunt (Figure 2(a)). Restriction fragment length polymorphism analysis confirmed that his father and aunt had this same mutation (Figure 2(b)). The patient's father and aunt had no history of any surgical procedures, although they developed the common cold at a typical frequency. Furthermore she had not become pregnant before then.

## 3. Discussion

We have presented a case of aHUS that developed in an infant after cardiac surgery for repair of tetralogy of Fallot. aHUS has been used to classify any HUS not caused by Shiga toxin. A variety of precipitating events have been associated with aHUS, including infections, drugs, autoimmune conditions, vaccination, malignancy, organ transplantation, pregnancy, and metabolic conditions [11, 12]. Although it is an uncommon postoperative complication, aHUS must be considered as a possible cause of acute kidney injury after surgical procedures [13]. Above all, the alternative complement pathway plays a key role in the pathogenesis of aHUS [11, 12]. Mutations in CFH account for approximately 25% of the genetic predisposition to aHUS [11, 14]. Mutations in CFI and MCP account for 5% to 10% and 10% of cases of aHUS, respectively [11, 15]. Mutations in C3 have been reported in several cohorts of patients with aHUS at a frequency of 4% to 10% [12, 16, 17].

Mutations of complement component C3 have been described more recently. C3 is cleaved to form the anaphylatoxins C3a and C3b, which are highly reactive and can bind to cell surfaces via their reactive thioester. C3b then can interact with CFB in the presence of factor D to form the alternative pathway of complement C3 convertase (C3bBb), which further cleaves C3, introducing a positive-feedback loop. Initial functional analysis showed that MCP was unable to bind to mutant C3, preventing its cleavage to iC3b [8]. Two C3 mutations that result in decreased secretion have been described, but their pathogenetic role remains uncertain. More recently, two mutations in C3 that bind to CFB with higher affinity and cause increased C3 convertase formation have been reported [18, 19]. These mutations result in increased complement activation on platelets [18] and the glomerular endothelium [19].

The underlying pathogenesis of TMA is considered to involve endothelial cell injury that results in renal arteriolar peritubular capillaries, intracapillary platelets, and fibrin-rich thrombi formation [20]. It is speculated that the pathogenesis of postoperative TTP involves massive endothelial damage during surgery [21]. In the present case, the endothelial cells might have been damaged during cardiac surgery. The presence of severe thrombocytopenia (platelet count ≤ 2.1 × 10^4^/*μ*L), anemia, and hemolytic parameters (elevated LDH and bilirubin levels) was observed immediately after surgery. These findings might be clues to the presence of aHUS that developed hyperacutely (Figure 1).

Fan et al. [22] recently reported the cause of aHUS in 10 Japanese patients. Eight cases were sporadic and the other two arose from one family. They identified 7 causative or potentially causative mutations in CFH (p.R1215Q), C3 (p.R425C, p.S562L, and p.I1157T), membrane cofactor protein (p.Y189D and p.A359V), and THBD (p.T500M) in 8 of the 10 patients. The patient with the n p.R425C mutation was our patient in the present report. Two mutations, p.R425C and p.S562L, are novel, and the p.I1157T mutation has been previously reported in the United States and Spain [16].

Fan et al. [22] described another patient with C3 mutation for whom surgery became the probable triggering event. The patient also had C3 p.I1157T, developed aHUS after undergoing nephrectomy at 70 years of age, and was treated with hemodialysis [23]. They stated that, in addition to the main genetic mutation, environmental factors and/or other genetic variations were likely required for the manifestation of aHUS as a secondary hit [23]. The cardiac surgery and/or presence of DIC might be secondary hits because other triggering events such as respiratory infection, diarrhea, aHUS-inducing drugs, and others were not demonstrated in our patient. Additionally, although our patient's father and aunt were affected by aHUS, they had heterozygous C3 mutation pR425C. This may be why they have not yet encountered a secondary hit.

As mentioned above, a variety of precipitating events are thought to contribute to the development of aHUS [11]. It is often reported that kidney transplantation is also a causative factor in aHUS [24]. However, there has been only one report describing the onset of aHUS initiated by a surgical procedure other than transplantation [13]. In that report, a 66-year-old woman developed renal impairment on the first day after laparoscopic hemicolectomy. aHUS is an uncommon postoperative complication; however, considering its different treatment modalities and poor outcomes, aHUS must be considered as a possible cause of acute kidney injury combined with thrombocytopenia and anemia after surgical procedures [13]. Conversely, TTP occurring in the nontransplantation postsurgical setting has been reported several times and has often been described after both cardiothoracic and vascular surgeries and noncardiovascular surgeries [25–28]. To the best of our knowledge, this is the first case report of postoperative aHUS due to a complement C3 mutation after nontransplantation surgery. In postsurgical patients who develop unexplained microangiopathic hemolytic anemia and thrombocytopenia, the diagnosis of aHUS should be considered and plasma exchange should be contemplated while other causes such as infection and disseminated intravascular coagulation are evaluated.

## Figures and Tables

**(a):** 

Parameters (unit of measurement)	−1POD	1POD	25POD	Normal range
White blood cell (/mm^3^)	14700	3700	29900	4000–9000
Hemoglobin (g/dL)	14.3	10.6	11.5	11.0–15.0
Platelet (×10^4^/mm^3^)	35.4	2.1	1.3	16.1–36.0
Albumin (g/dL)	4.8	3.0	2.8	3.9–4.9
AST (U/L)	73	294	126	12–29
ALT (U/L)	38	25	8	5–29
LDH (U/L)	292	1167	2147	106–220
Total bilirubin (mg/dL)	0.62	2.31	2.07	0.4–1.3
BUN (mg/dL)	9	9	108	9–21
Creatinine (mg/dL)	0.18	0.33	1.18	0.80–1.30
Haptoglobin (mg/dL)			<10	19–170
Schizocyte			+	(−)
PT-INR	1.03		2.33	1.0
APTT (s)	19.9		46.0	20–30
FDP (*μ*g/mL)	2		42	0–5

**(b):** 

Parameters (unit of measurement)	Results	Normal range
C3 (mg/dL)	40.0	86–160
C4 (mg/dL)	12.7	17–45
CH50 (IU/mL)	19.5	35–45
CFH gene mutation	Not detected	Not detected
CFI gene mutation	Not detected	Not detected
CFB gene mutation	Not detected	Not detected
MCP gene mutation	Not detected	Not detected
C3 gene mutation	R425C	Not detected
THBD gene mutation	Not detected	Not detected
Anti-FH antibody	Not detected	Not detected
ADAMTS-13 activity assay	Normal	Normal
